# Selective Plasma Mode Modulation in HfO_2_ Charge Trap Layers via RP/DP/RP Atomic Layer Deposition for Enhanced Memory Performance and Interface Quality

**DOI:** 10.3390/mi17080965

**Published:** 2026-08-15

**Authors:** Byungwook Kim, Yongwoon Jang, Hyeonwu Nam, Changyun Hong, Minkyun Kang, Wookyung Lee, Changbun Yoon

**Affiliations:** Department of Advanced Materials Engineering, Tech University of Korea, Siheung-si 15073, Republic of Korea; quddnr000@tukorea.ac.kr (B.K.); jyw5414@tukorea.ac.kr (Y.J.); hyeonwu0604@tukorea.ac.kr (H.N.); jake3121@tukorea.ac.kr (C.H.); dakldsa@tukorea.ac.kr (M.K.); ds090390@tukorea.ac.kr (W.L.)

**Keywords:** hafnium oxide, plasma-enhanced atomic layer deposition, charge trap memory (CTM), remote/direct plasma modulation, memory window

## Abstract

Hafnium oxide (HfO_2_) has emerged as a promising charge trap layer (CTL) for nonvolatile memory devices; however, its long-term reliability remains challenging due to leakage current through grain boundary pathways and interface defect formation. Within plasma-enhanced atomic layer deposition (PEALD), direct plasma ALD (DPALD) forms crystalline HfO_2_ with deeper trap sites, while its ion-assisted nature may contribute to interface damage, resulting in a lower net trap density than remote plasma ALD (RPALD), which better preserves interfacial quality. We propose an interface–bulk–interface (RP/DP/RP) process design that places RPALD at the interfaces and DPALD in the bulk, aiming to combine bulk trap sites with interfacial protection. Charge trap memory (CTM) devices were fabricated across RP/DP/RP thickness combinations. Within the tested range, the optimized structure exhibited the widest memory window and the highest effective trapped charge density among the measured devices, improving upon DPALD and comparable to RPALD, together with the lowest near-interface trap density at the Si/Al_2_O_3_ interface. The device also maintained a stable memory window up to 10^4^ program/erase cycles, and short-term retention data suggest potentially stable program and erase states, requiring longer-term validation. These results indicate that spatial modulation of plasma modes offers a process-level strategy for improving HfO_2_-based CTM devices.

## 1. Introduction

As semiconductor devices continue to evolve toward high-density vertical integration, scaling of the charge trap layer (CTL) has emerged as a critical challenge, requiring deposition processes compatible with extremely high-aspect-ratio (HAR) structures [1,2,3,4]. Conventional silicon nitride (SiN)-based CTLs offer well-established process maturity; however, reducing the film thickness leads to degradation in multi-bit storage reliability, including a decreased trap charge density (N_t_), a reduced memory window (MW), and an increased leakage current [5]. Furthermore, the inherently shallow trap depth and broad energy distribution of trap states in SiN fundamentally limit long-term charge retention, posing additional constraints on further scaling. To overcome these limitations, high-k oxide-based CTLs have been proposed as promising alternatives. Among these materials, hafnium oxide (HfO_2_) has attracted considerable attention as a potential replacement for SiN because its deeper trap energy levels, larger band offset with silicon, lower equivalent oxide thickness (EOT), and compatibility with complementary metal–oxide–semiconductor (CMOS) processes provide a more favorable combination of charge trapping capability and scalability than conventional nitride-based layers [6,7,8,9,10].

However, pristine HfO_2_ thin films are prone to charge loss and reliability degradation associated with intrinsic defects such as oxygen vacancies and limited thermal stability [11,12]. To mitigate these drawbacks, material-based approaches, including metal-element doping [13], nanocrystal incorporation [14,15], and post-deposition annealing (PDA) [16], have been extensively investigated. However, these strategies are often accompanied by increased process complexity and elevated thermal budget constraints. Consequently, growing interest has shifted toward process-oriented strategies based on plasma-enhanced atomic layer deposition (PEALD), which enables independent control of interfacial and bulk properties without altering the material composition [17,18].

Among the various PEALD configurations, plasma mode modulation has emerged as a particularly promising strategy, as it allows spatial control of film properties within a single deposition sequence. Furthermore, the low processing temperature and precise thickness control of PEALD make it particularly suitable for deposition in the HAR structures required for 3D memory devices [19,20,21]. PEALD is broadly classified into direct plasma atomic layer deposition (DPALD) and remote plasma atomic layer deposition (RPALD) based on the plasma configuration [22]. DPALD promotes the formation of crystalline HfO_2_ via energetic ion–surface interactions; the resulting polycrystalline phase is associated with deeper trap energy levels [23,24,25].

However, the accompanying ion bombardment has been reported to concurrently generate interface trap states that can hinder efficient charge trapping and de-trapping through the tunnel oxide—this effect is considered a major contributor to the reduced net effective trap density observed in single-scheme DPALD films [26,27,28]. RPALD, by spatially separating the plasma discharge from the substrate, is expected to mitigate ion bombardment and typically produces amorphous HfO_2_ with improved interfacial stability, which has been associated with a higher net trap density and improved charge retention [29,30,31,32]. These two schemes are thus considered complementary.

In this study, exploiting this complementarity and considering that both the tunnel oxide and blocking oxide interfaces may be susceptible to ion-induced damage, we propose an interface–bulk–interface (RP/DP/RP) CTL process design. RPALD is applied at the tunnel-oxide interface primarily to mitigate defect formation and support efficient charge trapping, and at the blocking-oxide interface to suppress leakage toward the top electrode, whereas DPALD is employed in the bulk region to promote charge trapping and MW enhancement. Charge trap memory (CTM) devices with a p-Si/Al_2_O_3_/HfO_2_/Al_2_O_3_/Al structure were fabricated, and the RP/DP/RP thickness ratio was systematically varied to identify the best-performing configuration within the tested range. The MW, near-interface trap density at the Si/Al_2_O_3_ interface (D_it_), endurance, and charge retention were evaluated to assess the effectiveness of the proposed process-level design strategy.

## 2. Materials and Methods

### 2.1. CTM Device Fabrication

**Figure 1:**
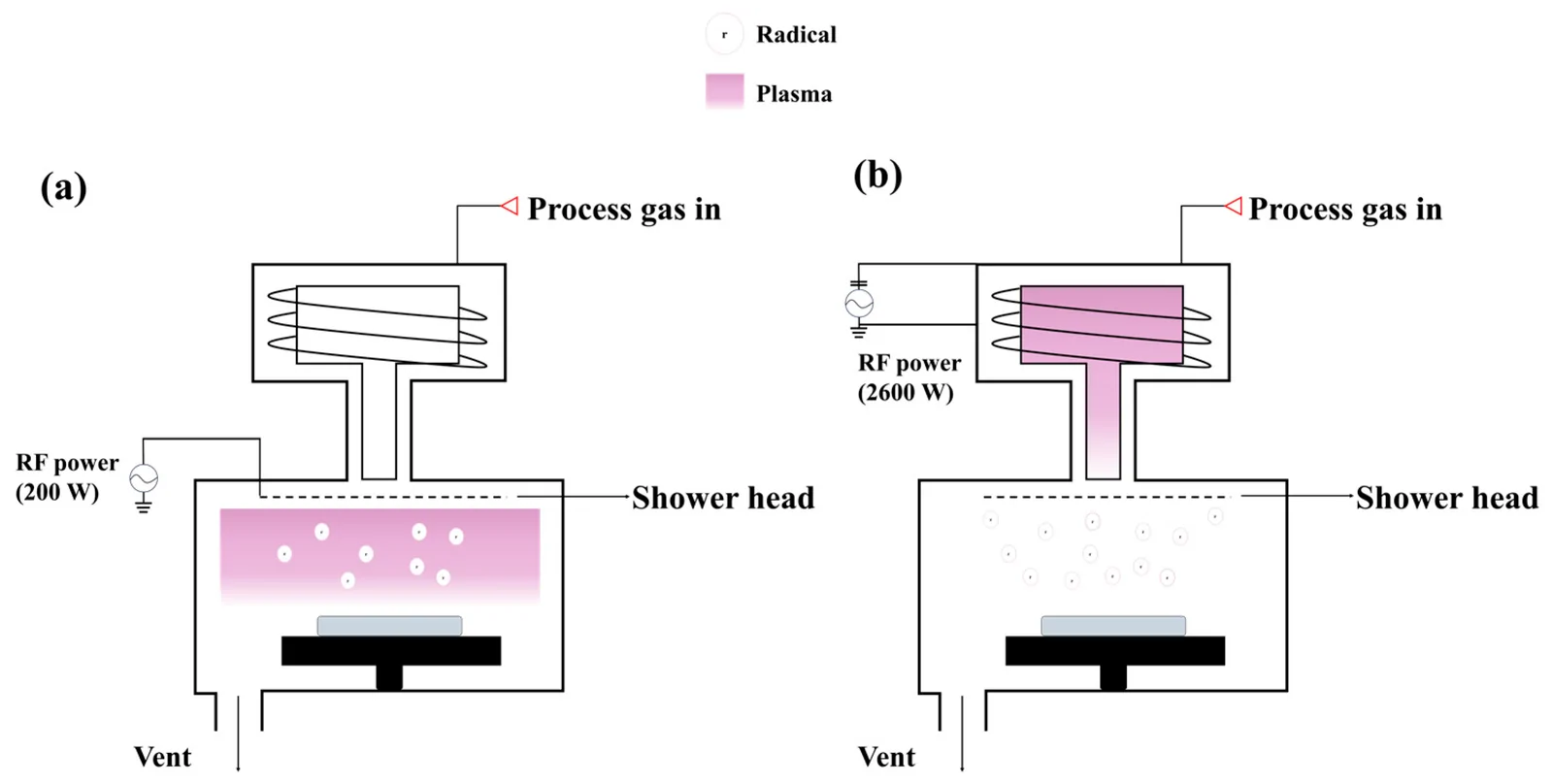
Schematic illustrations of the deposition mechanisms of (**a**) DPALD and (**b**) RPALD processes.

The schematics of the DPALD and RPALD processes are shown in Figure 1. The PEALD system used in this study was iOV-dx2 (iSAC Research, Daejeon, Republic of Korea). Tetrakis(ethylmethylamido)hafnium (TEMA-Hf) and trimethylaluminum (TMA), both supplied by iChems (Hwaseong, Republic of Korea), served as the precursors for HfO_2_ and Al_2_O_3_, respectively. In the DP mode (Figure 1a), the substrate was placed inside the reaction chamber and exposed to a 200 W capacitively coupled plasma generated directly within the chamber. In the RP mode (Figure 1b), an RPS unit (En2ra-RPS, EN2CORE Technology, Daejeon, Republic of Korea) was integrated into the same PEALD chamber, such that the reaction chamber, gas lines, and precursor supply were shared between the two modes while spatially separating the plasma generation region; the predominantly activated oxygen radicals derived from a 2600 W inductively coupled plasma source were supplied to the substrate surface. A single ALD cycle for HfO_2_ thin film deposition consisted of four sequential steps: (1) Hf precursor exposure, (2) Ar purge, (3) plasma exposure (O_2_ gas), and (4) Ar purge. For the DP process, the precursor exposure time, purge time, plasma exposure time, and purge time were set to 5, 10, 2, and 20 s, respectively. The short plasma exposure of the DP process was chosen within the self-limited growth window to minimize direct ion exposure to the growth surface. For the RP process, the corresponding times were set to 5, 10, 15, and 30 s, respectively. The higher source power (2600 W) and longer plasma exposure of the RP process were employed to secure a sufficient radical density at the substrate, since the radicals generated in the remote unit undergo recombination during transport to the reaction chamber. While the chamber, gas lines, and precursor supply were shared, the plasma power, exposure time, and resulting plasma dose, radical flux, and ion-energy distribution still differed substantially between DP and RP, so the comparison isolates the plasma configuration rather than a single variable alone.

**Figure 2:**
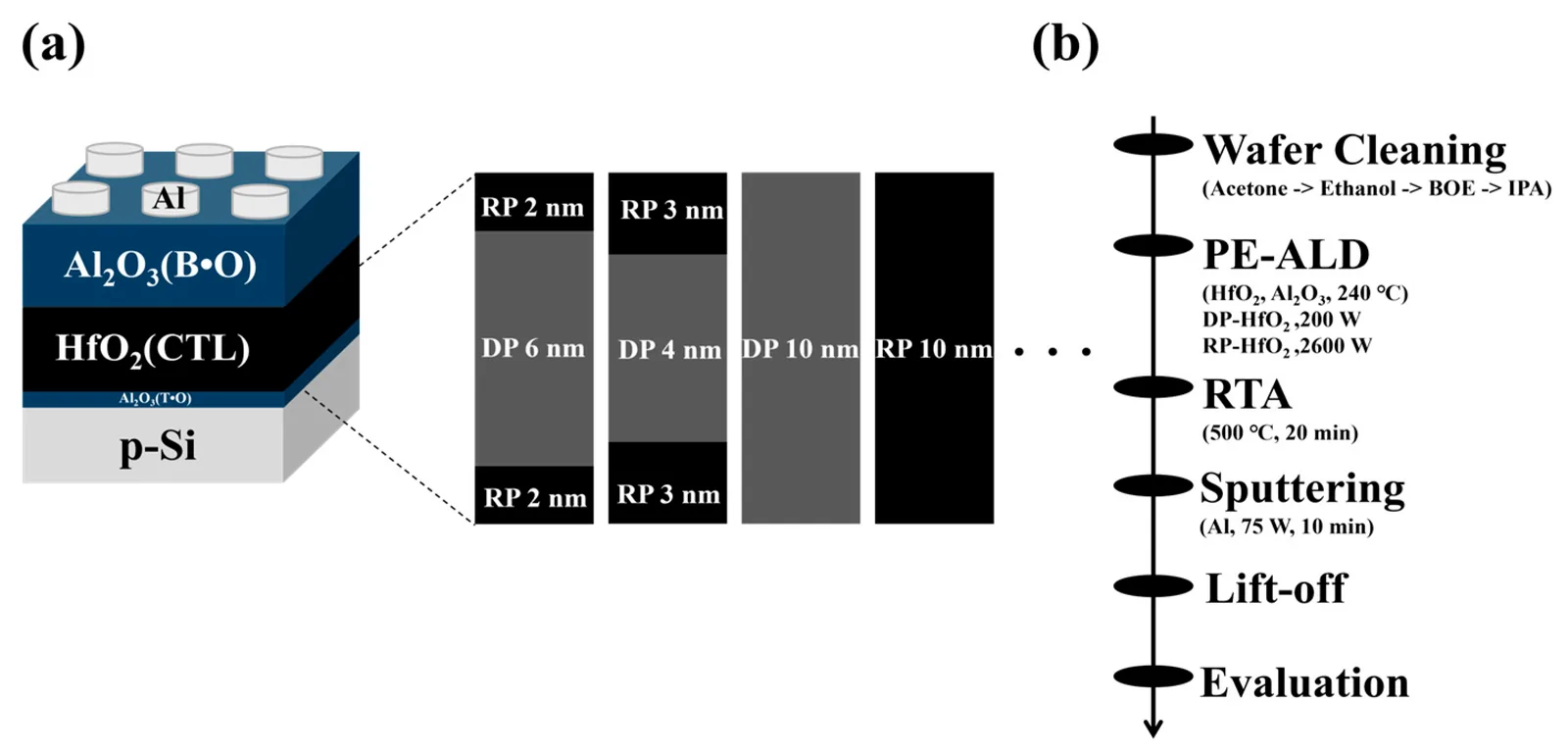
(**a**) Schematic of the CTM device structure with an HfO_2_ CTL. (**b**) Schematic of the fabrication process for the HfO_2_ CTL.

The fabrication strategy and process flow of the CTM devices are shown in Figure 2. As shown in Figure 2a, the device structure comprises a p-Si substrate/Al_2_O_3_ tunneling oxide (2 nm)/HfO_2_ CTL (10 nm)/Al_2_O_3_ blocking oxide (10 nm)/Al top electrode (100nm) stack. The HfO_2_ CTL was fabricated by systematically varying the RP/DP/RP thickness combinations, denoted as 1/8/1, 2/6/2, 3/4/3, and 4/2/4 nm based on the target thickness of each sublayer, while maintaining a total target thickness of 10 nm. DP (10 nm) and RP (10 nm) single-process reference samples were also prepared for comparison. According to the process flow in Figure 2b, the p-Si substrates were sequentially cleaned with acetone, ethanol, buffered oxide etchant, and isopropanol. Thin films (Al_2_O_3_ and HfO_2_) were then deposited at 240 °C using the same PEALD system described above. Following deposition, all samples underwent PDA at 500 °C for 20 min in an N_2_ atmosphere using a rapid thermal annealing system (RTA700, PyroTec, Gyeonggi-do, Republic of Korea). This thermal treatment was employed to promote partial crystallization of the film, which is expected to introduce structural features such as oxygen-related defects and grain boundaries that may contribute to charge trapping. Subsequently, Al top electrodes (100 nm thick) were deposited by DC magnetron sputtering at 75 W for 10 min using a sputtering system (BLS, Gyeonggi-do, Republic of Korea), and circular electrode patterns with a diameter of 200 μm were defined via a lift-off process to complete the device fabrication.

### 2.2. Thin Film Characterization and Electrical Evaluation of HfO_2_ CTM Devices

The thicknesses of the HfO_2_ thin films were measured using a spectroscopic ellipsometer (Elli-SE-U, Ellipso Technology, Suwon-si, Republic of Korea). The crystallographic structure and phase characteristics of the HfO_2_ thin films were analyzed by grazing-incidence X-ray diffraction (GI-XRD; SmartLab, Rigaku, Tokyo, Japan). The chemical bonding states of the HfO_2_ films were analyzed by X-ray photoelectron spectroscopy (XPS; AXIS SUPRA+, Kratos Analytical, Manchester, UK) at the Advanced Institute of Convergence Technology, using a monochromated Al Kα source (1486.6 eV). The surface morphology and roughness of the HfO_2_ thin films deposited under DP and RP conditions were evaluated using atomic force microscopy (AFM; XE150, Park Systems, Suwon-si, Republic of Korea). The cross-sectional structure and thickness of the HfO_2_ thin films were characterized by high-resolution transmission electron microscopy (HR-TEM; Tecnai G2 F20, FEI, Hillsboro, OR, USA). The electrical characteristics of the fabricated memory devices were evaluated using a semiconductor parameter analyzer (4200A-SCS, Keithley Instruments, Cleveland, OH, USA) connected to a probe station (APX-6B, WIT Co., Suwon-si, Republic of Korea). Unless otherwise noted, three devices were measured for each condition, and results are reported as the mean standard deviation; for Weibull breakdown analysis, fifteen devices per condition were measured to ensure a statistically reliable distribution. C–V measurements were performed at 1 MHz with a 100 mV AC signal amplitude, sweeping the gate voltage slowly from negative to positive bias with no additional delay time; the MW was determined as the horizontal separation between the program and erase hysteresis curves at a fixed capacitance level within the depletion region. J–V measurements were performed with a 0.1 V voltage step at a slow sweep rate, with the negative- and positive-bias regions measured separately under a current compliance of 0.042 A; the breakdown voltage was defined as the voltage at which the measured current reached 10^−3^ A, beyond which the device was permanently and destructively damaged, and current density was calculated by normalizing the measured current to the effective electrode area (3.14 × 10^−4^ cm^2^, 200 μm diameter). The trap density (N_t_) and interface-state density (D_it_) near the Al_2_O_3_/Si interface were both evaluated from the C–V characteristics: N_t_ was obtained from the saturated MW value, and D_it_ was extracted from the 1 MHz C–V curve using a voltage-difference method based on the stretch-out of the measured curve relative to an ideal (trap-free) curve. The ideal C–V curve was generated using the nanoHUB MOSCap tool [33] (p-Si, N_A_ ≈ 1 × 10^15^ cm^−3^), with C_ox_ set as the accumulation capacitance.

## 3. Results

### 3.1. Structural and Chemical Characterizations of HfO_2_ CTM Devices

**Figure 3:**
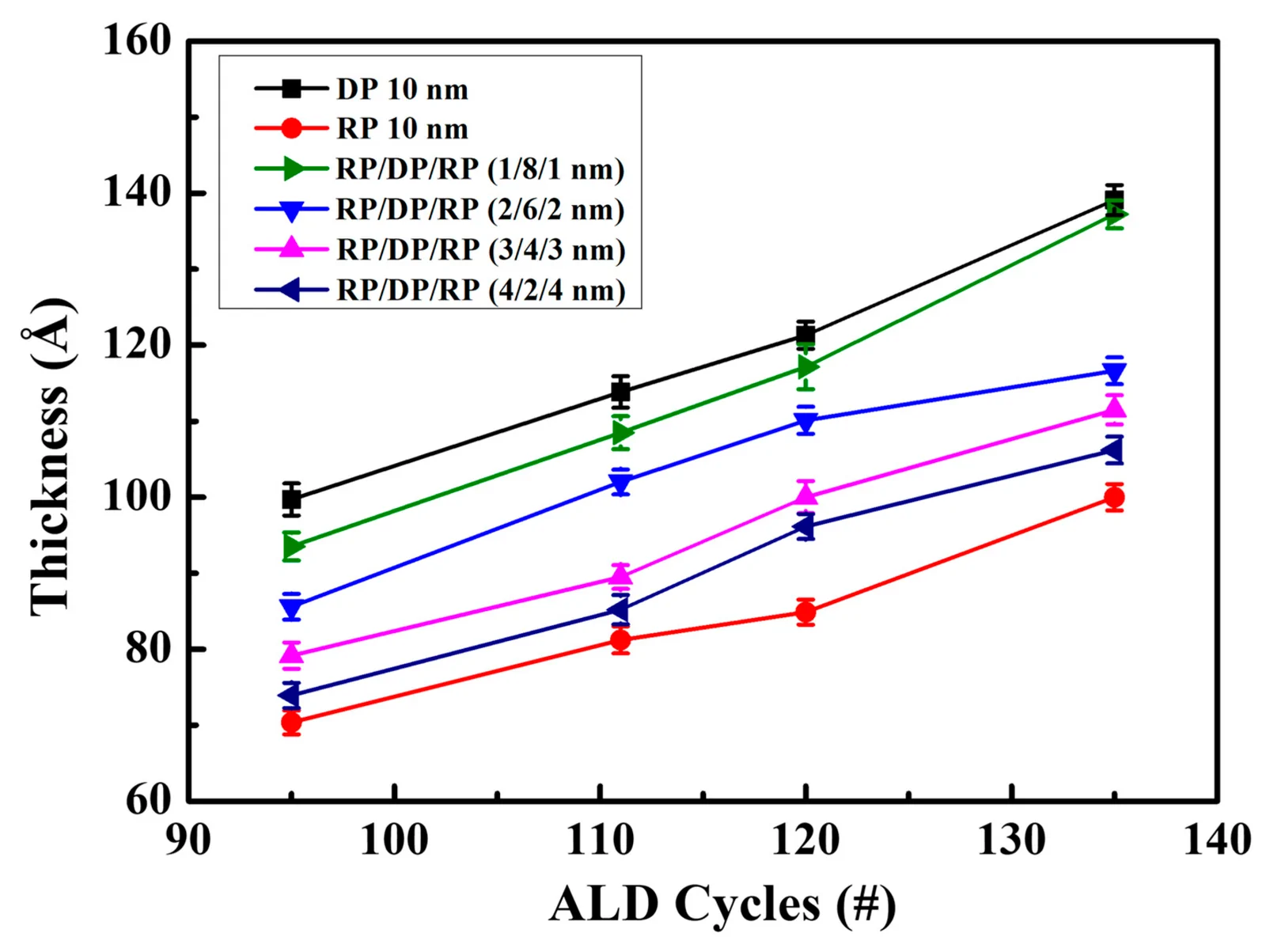
HfO_2_ CTL thickness as a function of ALD cycle number (0–135 cycles) for the DPALD, RPALD, and modulated RP/DP/RP sequences, used to extract the growth-per-cycle (GPC) value of each condition via linear fitting.

Figure 3 shows the thickness of HfO_2_ CTLs as a function of ALD cycles for the DPALD, RPALD, and modulated RP/DP/RP stacking sequences. This common cycle range (0–135 cycles) was applied identically across all sequences to extract a reliable GPC value for each condition from linear fits to the thickness–cycle data, rather than to represent the cycle counts used for the fabricated devices. In all cases, the film thickness increased nearly linearly with the number of cycles, indicating that linear growth behavior consistent with the self-limited surface reaction chemistry of ALD [34] was maintained for both the single-mode and plasma-modulated (cross-deposited) sequences, and the error bars at each data point support process reproducibility. The GPC values were extracted from linear fits to the thickness–cycle data for each deposition process. The DPALD process exhibited the highest GPC of 1.05 Å/cycle, reflecting enhanced growth efficiency attributed to ion-assisted reactions. In contrast, the RPALD process showed the lowest GPC of 0.74 Å/cycle, attributed to the radical-driven reaction mechanism.

For the modulated RP/DP/RP structures, the GPC increased monotonically with increasing DPALD fraction. The 1/8/1 nm structure, which has the highest DPALD fraction, exhibited a GPC of 0.97 Å/cycle, approaching that of DPALD. As the DPALD fraction decreased, the GPC decreased sequentially to 0.90 Å/cycle for the 2/6/2 nm structure, 0.83 Å/cycle for the 3/4/3 nm structure, and 0.78 Å/cycle for the 4/2/4 nm structure, converging toward the RPALD value. At 135 cycles, the common endpoint used for this GPC calibration, the DPALD (~139 Å) and 1/8/1 nm (~137 Å) structures yielded the thickest films, whereas the 4/2/4 nm (~106 Å) and RPALD (~100 Å) structures produced comparatively thinner films. Using these GPC values, the cycle allocation required to reach the 10 nm target thickness was calculated separately for each structure, as summarized in Table 1. Spectroscopic ellipsometry showed that the measured total thicknesses of all fabricated structures fell within a narrow range of 9.54–10.1 nm regardless of the RP/DP/RP cycle ratio, suggesting that the GPC-based cycle allocation reliably reproduced the intended 10 nm HfO_2_ CTL thickness across all structures.

These trends indicate that the overall GPC of the RP/DP/RP architecture can be predictably tuned by adjusting the DPALD/RPALD thickness ratio, enabling precise control of the final film thickness.

**Figure 4:**
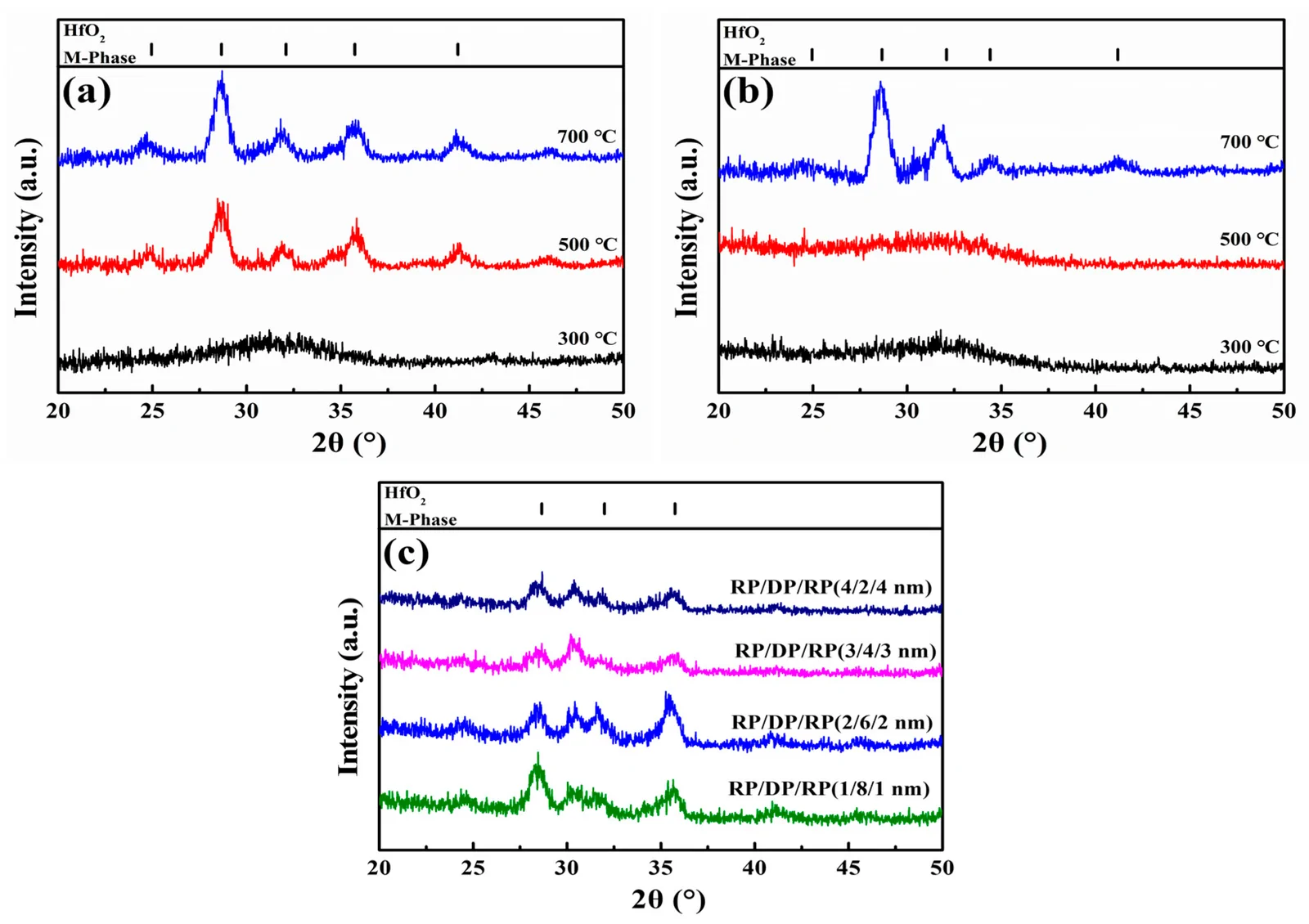
GI-XRD patterns of HfO_2_ CTLs deposited using (**a**) DPALD and (**b**) RPALD processes, annealed at 300, 500, and 700 °C, and (**c**) modulated RP/DP/RP structures annealed at 500 °C.

The GI-XRD patterns of HfO_2_ films deposited by DPALD and RPALD and annealed at 300, 500, and 700 °C are shown in Figure 4a and Figure 4b, respectively. Under the 300 °C annealing condition, both processes yielded amorphous films with no discernible diffraction peaks. As the annealing temperature increased, all samples developed crystalline diffraction features, with reflections appearing at positions consistent with those reported for crystalline HfO_2_ [35]. At 700 °C, the DPALD film showed well-defined diffraction peaks near 2θ ≈ 28°, 31°, and 36°, whereas the RPALD film exhibited relatively weaker and broader diffraction features, primarily near 2θ ≈ 28°. The diffraction peaks near 31° and 36° were indistinct, which is consistent with a lower degree of crystallization. Peak height alone was not used as a measure of crystallinity, since diffraction intensity also depends on film thickness, preferred orientation, and measurement geometry; all films compared here had comparable total thicknesses (9.54–10.1 nm, Table 1) and were measured under identical grazing-incidence conditions. A notable difference in crystallization behavior was observed between DPALD and RPALD. As shown in Figure 4a, the DPALD film began to exhibit distinct crystalline peaks near 2θ ≈ 28°, 31°, and 36° upon annealing at 500 °C. This difference may be attributed to the ion bombardment during DPALD, which is expected to promote crystallization at lower annealing temperatures by providing additional energy for atomic rearrangement. In contrast, the RPALD film shown in Figure 4b remained amorphous even at 500 °C. Because 500 °C represents a temperature regime in which the DPALD film crystallizes while the RPALD film remains amorphous, it was selected as the annealing condition in this work. This selection was based on the structural crystallization behavior rather than on a direct comparison of electrical characteristics across annealing temperatures. Under this condition, the crystalline phase is expected to contribute to trap-site formation, while the retained amorphous phase may suppress grain boundary leakage current.

The GI-XRD patterns of the four modulated RP/DP/RP structures (1/8/1, 2/6/2, 3/4/3, and 4/2/4 nm) annealed at 500 °C are presented in Figure 4c. All modulated structures exhibited broader and weaker diffraction features than the DPALD film, while remaining clearly distinguishable from the featureless pattern of the amorphous RPALD film. This indicates that the modulated stacks develop a partially crystallized state intermediate between the two single-mode references. Across the series, the integrated intensity of the diffraction features decreased as the RPALD interfacial fraction increased, suggesting that the RPALD regions restrict grain growth within the stack. Since all structures had comparable total thicknesses and were measured under identical conditions, this trend is presented as a relative comparison rather than an absolute measure of crystallinity. These results suggest that the degree of crystallization of the HfO_2_ CTL can be tuned through the RP/DP/RP thickness combination without any change in material composition, which supports the modulated deposition approach as a process-level route to controlling the structural state of the charge trapping layer. The reduced crystallization observed at higher RPALD fractions may in turn reduce grain-boundary leakage pathways, which is consistent with the leakage-current behavior described in Section 3.2.

**Figure 5:**
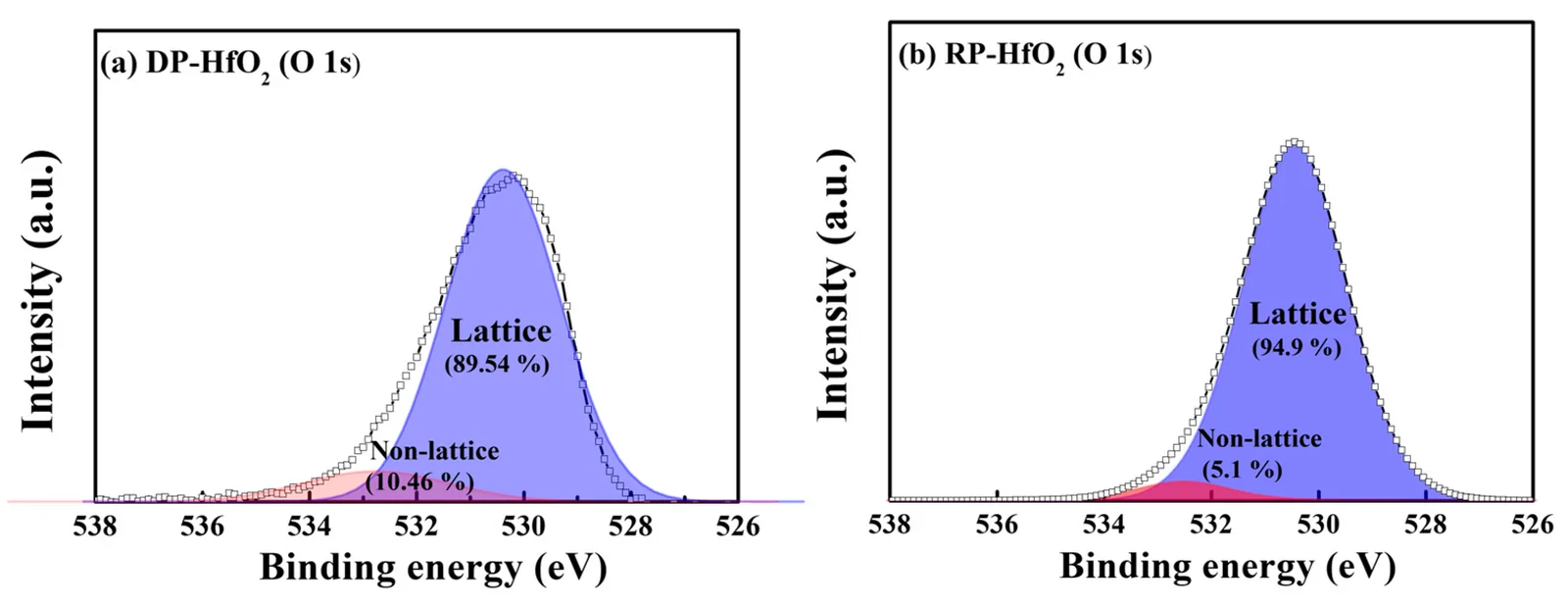
O 1s XPS spectra of HfO_2_ films deposited by (**a**) DPALD and (**b**) RPALD processes. All binding energies were referenced to the adventitious C 1s peak at 284.8 eV, and a Shirley background was applied. The spectra were deconvoluted into lattice-oxygen and non-lattice-oxygen components using pseudo-Voigt line shapes under identical fitting constraints for both films.

Figure 5 shows the O 1s XPS analysis of the HfO_2_ films deposited by DPALD and RPALD. In both films the spectra are dominated by the lattice-oxygen component, with a non-lattice oxygen component—commonly associated with oxygen vacancies—appearing on the higher-binding-energy side. The relative proportion of non-lattice bonding was larger for the DPALD film than for the RPALD film. This suggests that the ion impact during the DP process may influence oxygen-vacancy formation in the film, whereas the reduced ion damage in the RP process may lead to a relatively lower degree of oxygen-defect formation.

**Figure 6:**
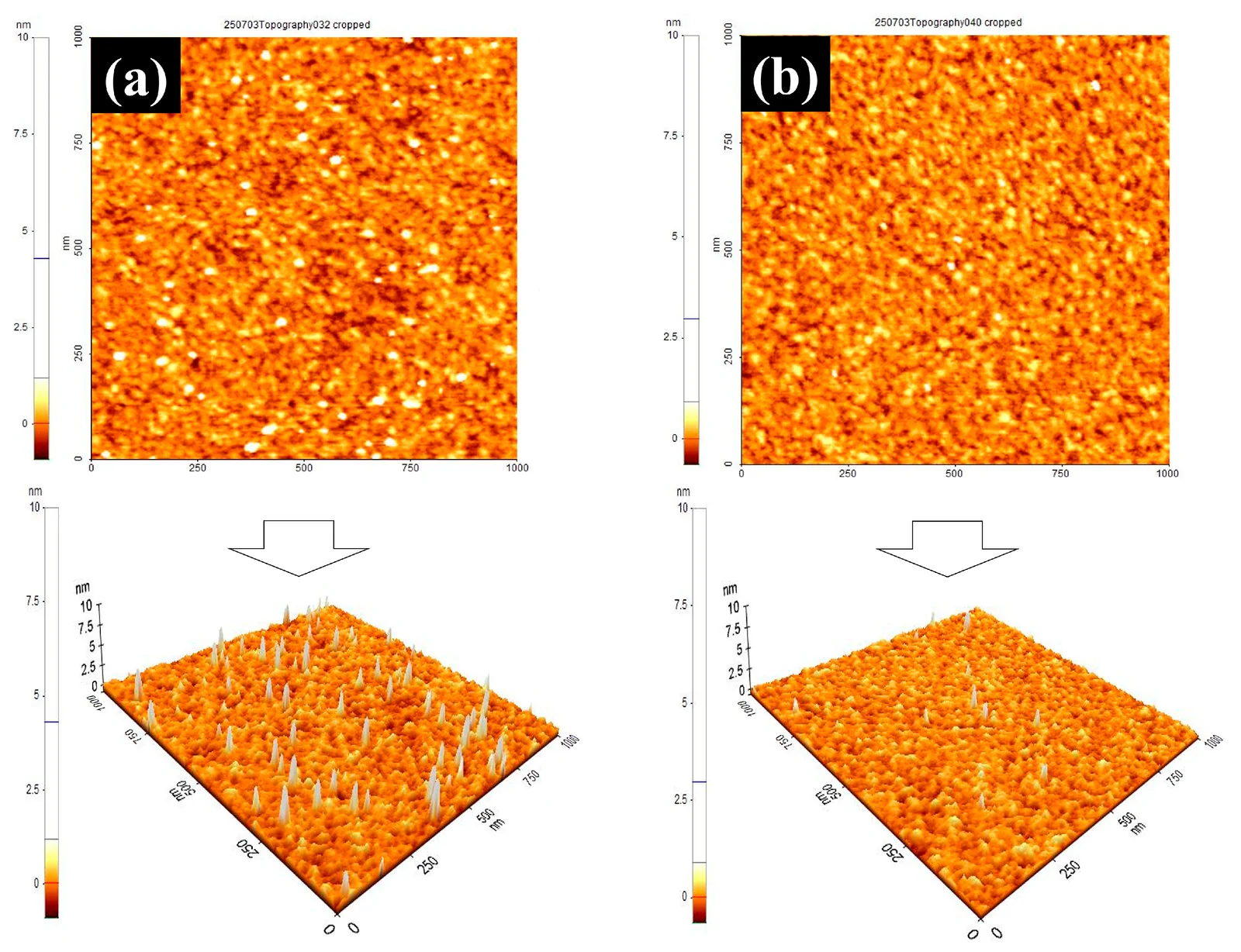
2D and 3D AFM surface images of HfO_2_ thin films deposited using (**a**) DPALD and (**b**) RPALD processes.

Figure 6 shows the 2D and 3D AFM surface images of HfO_2_ thin films deposited by DPALD and RPALD, along with their quantitative roughness analysis. As shown in Figure 6a, the DPALD film exhibited a nonuniform distribution of bright spots in the 2D height map, indicating locally elevated surface features. The corresponding 3D image revealed protruding hillock-like features with significant height variation across the scanned area. These protrusions are qualitatively consistent with localized grain growth that may be promoted by the ion-assisted nature of the DP process. Quantitative analysis yielded a mean roughness (Ra) of 0.203 nm and a root-mean-square roughness (Rq) of 0.354 nm. In contrast, the RPALD film shown in Figure 6b exhibits a uniform color distribution in the 2D image and a markedly smoother surface morphology in the 3D image, with substantially fewer protrusions compared to the DPALD film. The Ra and Rq values were measured to be 0.137 nm and 0.183 nm, respectively, corresponding to reductions of approximately 32.5% and 48.3% relative to those of the DPALD film. These differences in surface roughness are consistent with the crystallinity contrast between the DPALD and RPALD films observed in the GI-XRD analysis and suggest that the predominantly radical-driven reaction pathway of RPALD is associated with reduced ion-induced surface modification, which may contribute to more uniform film growth [36].

**Figure 7:**
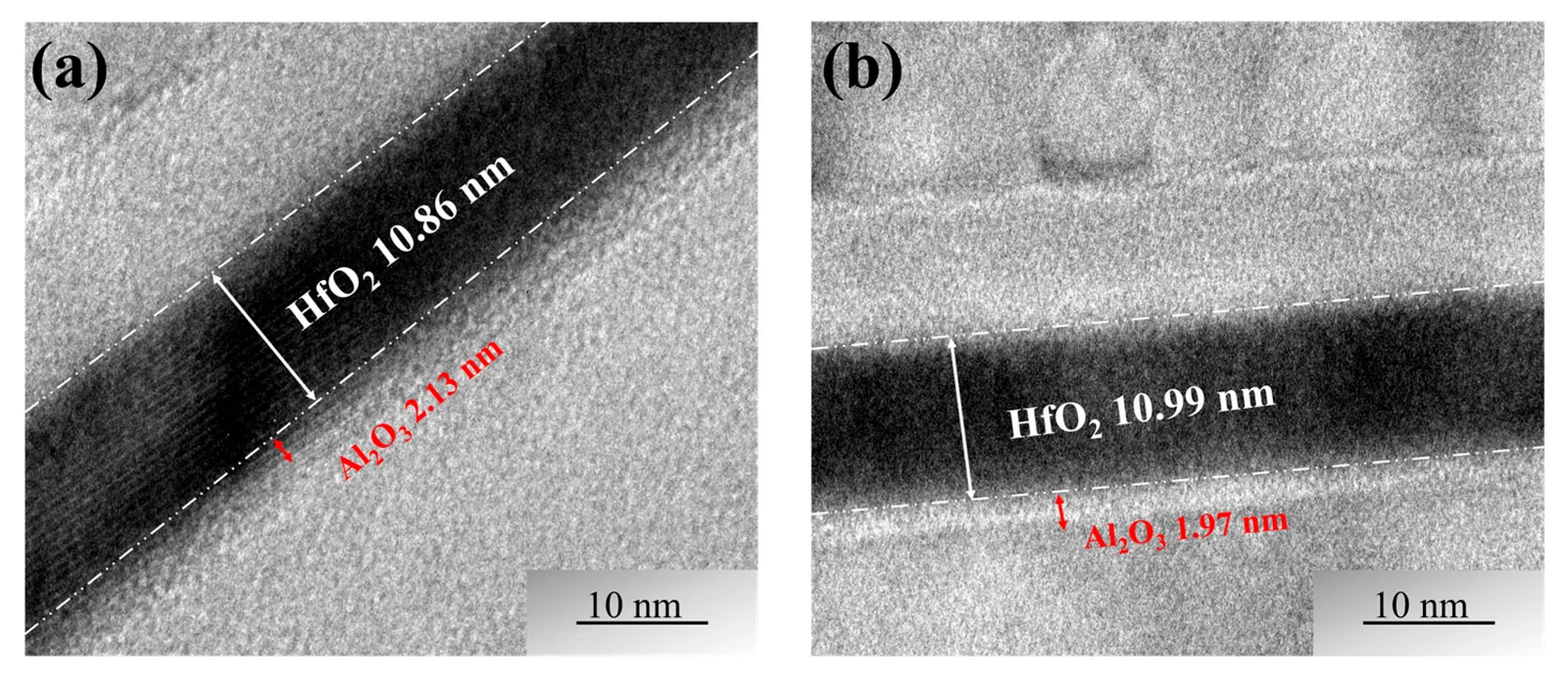
Cross-sectional TEM images of (**a**) the DPALD device and (**b**) the RP/DP/RP (3/4/3 nm) device.

Figure 7 shows the cross-sectional HR-TEM images of the DPALD and RP/DP/RP (3/4/3 nm) devices, providing direct structural characterization of the layer thicknesses and interfacial quality. As shown in Figure 7a, the HfO_2_ and Al_2_O_3_ layer thicknesses in the DPALD device were measured to be approximately 10.86 nm and 2.13 nm, respectively, with a well-defined interface observed between the two layers. The RP/DP/RP (3/4/3 nm) device shown in Figure 7b exhibited comparable layer thicknesses of approximately 10.99 nm for HfO_2_ and 1.97 nm for Al_2_O_3_, indicating that the modulated RP/DP deposition sequence does not noticeably degrade the thickness uniformity of the final stacked structure. The boundary between the RP and DP sublayers within the HfO_2_ CTL is not visually discernible in the HR-TEM image, which is consistent with a structurally continuous film; however, because HR-TEM contrast alone cannot resolve compositionally identical sublayers, this does not by itself exclude the intended RP/DP/RP sublayer structure.

Further inspection of both HR-TEM images reveals clearly resolved periodic lattice fringes in localized regions of the HfO_2_ layers. The measured d-spacing values of approximately 3.15 Å and 2.83 Å are consistent with the values theoretically estimated from Bragg’s law (λ = 2dsinθ, λ= 1.5406 Å) utilizing the dominant diffraction peaks at 2θ ≈ 28° and 31° in the XRD patterns, which may be assigned to the (−111) and (111) planes of monoclinic HfO_2_, respectively. These localized fringes indicate the presence of crystalline regions but are not a quantitative measure of the overall crystallinity, which was instead evaluated by GI-XRD (Figure 4). In contrast, the Al_2_O_3_ layer and the adjacent substrate region exhibit a disordered, featureless contrast, consistent with an amorphous nature. Furthermore, no distinct interfacial diffusion layer was observed in the HR-TEM contrast between the HfO_2_ and Al_2_O_3_ layers; however, as conventional TEM contrast does not provide direct chemical information, this observation is not a quantitative exclusion of interdiffusion.

### 3.2. Electrical Characteristics of HfO_2_ CTM Devices

**Figure 8:**
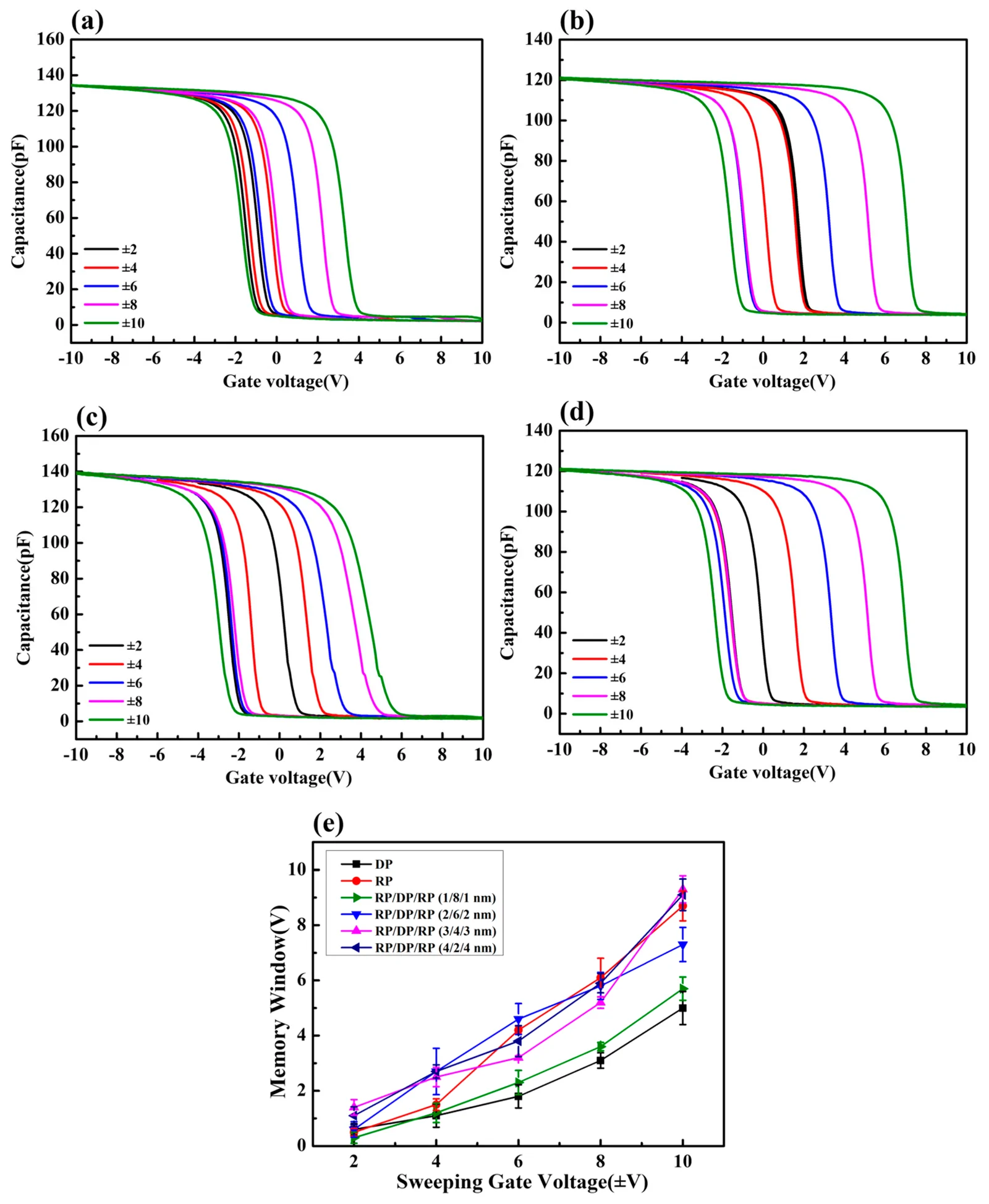
C–V hysteresis curves of HfO_2_ CTM devices fabricated using (**a**) DPALD, (**b**) RPALD, (**c**) RP/DP/RP (2/6/2 nm), and (**d**) RP/DP/RP (3/4/3 nm) structures, and (**e**) MW as a function of sweeping gate voltage for all devices.

The high-frequency (1 MHz) capacitance–voltage (C–V) hysteresis curves of HfO_2_ CTM devices fabricated using DPALD, RPALD, and modulated stacking sequences are shown in Figure 8a–d. Representative C–V curves are presented for the two single-process references (DPALD and RPALD) and two representative modulated structures (2/6/2 and 3/4/3 nm), while the memory windows of all six structures are summarized in Figure 8e. The measurements were performed by progressively increasing the gate-sweep voltage from ±2 V to ±10 V. As shown in Figure 8a–d, all devices exhibited well-defined hysteresis loops, the width of which increased with increasing sweep voltage.

To quantitatively evaluate the charge-storage capability of each device, the MW was plotted as a function of gate-sweep voltage in Figure 8e. The error bars represent the standard deviation over three devices per condition, suggesting reasonable reproducibility and uniformity of the fabrication process. Under ±10 V operation, the DPALD device shown in Figure 8a exhibited the lowest MW of 5.0 V, whereas the RPALD device shown in Figure 8b exhibited a notably improved MW of 8.7 V. Among the modulated structures, the RP/DP/RP (1/8/1 nm) and (4/2/4 nm) devices yielded MW values of 5.7 V and 9.1 V, respectively. The RP/DP/RP (2/6/2 nm) device shown in Figure 8c yielded an MW of 7.3 V, while the RP/DP/RP (3/4/3 nm) device shown in Figure 8d yielded an MW of 9.3 V.

Analysis of the MW–sweep voltage characteristics in Figure 8e reveals a common trend of increasing MW with increasing sweep voltage across all devices. The MW increased systematically with the RP interfacial fraction: the DPALD and RP/DP/RP (1/8/1 nm) devices, with the smallest RP fraction, showed lower MW, whereas the (2/6/2), (3/4/3), and (4/2/4 nm) devices reached MW values comparable to that of the RPALD reference. At ±10 V, the (3/4/3 nm), (4/2/4 nm), and RPALD devices differed by less than ~0.6 V, which is within the device-to-device variation indicated by the error bars; the modulated devices therefore attain RPALD-level charge storage rather than exceeding it. In contrast, the DPALD device shows MW values comparable to the other devices at low sweep voltages (±2–4 V), but a reduced rate of MW increase at higher voltages, which may be associated with interface damage related to the ion-assisted DP process, potentially limiting charge injection at higher fields. These results suggest that incorporating RPALD interfacial layers in the modulated RP/DP/RP scheme contributes to improved charge trapping behavior, which may be related to a higher effective trapped charge density within the HfO_2_ CTL [37].

**Figure 9:**
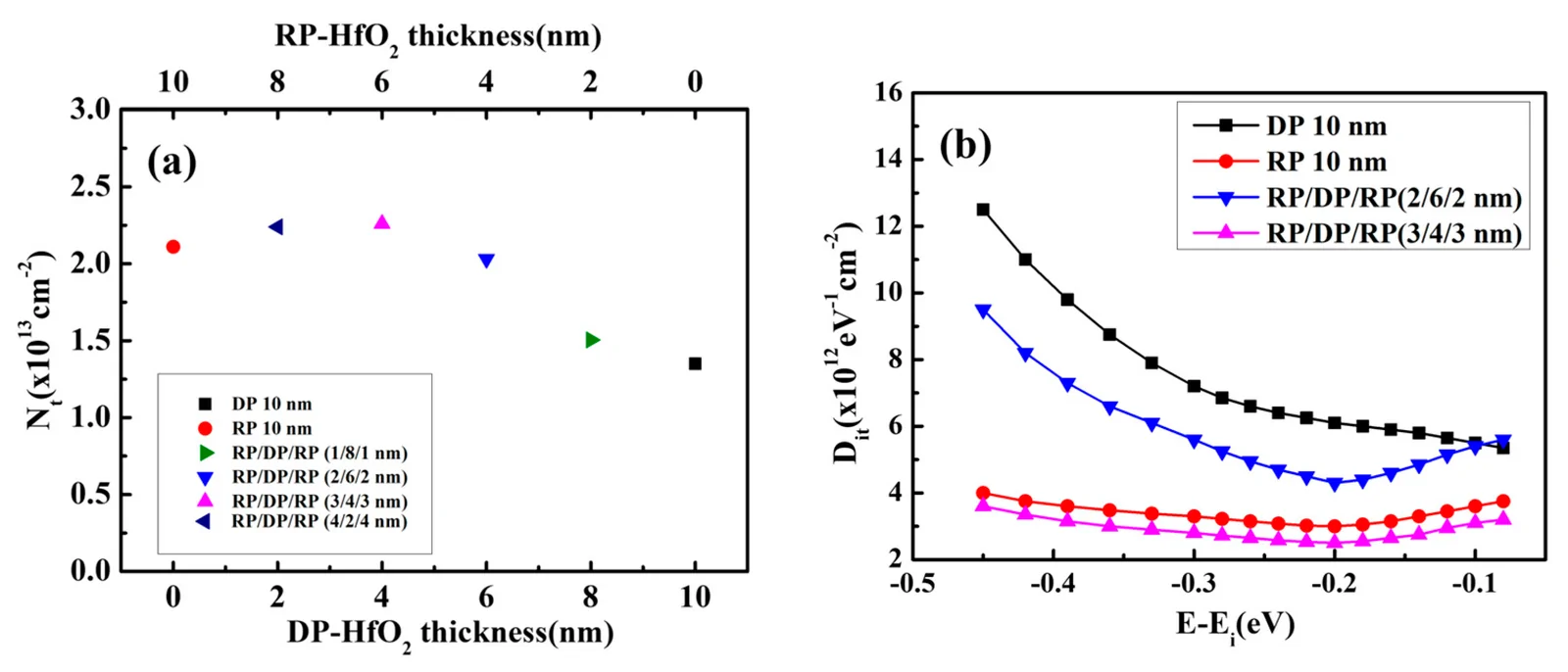
Defect analysis of HfO_2_ CTM devices: (**a**) effective trapped charge density (N_t_) and (**b**) interface trap density (D_it_).

To further examine the factors underlying the enhanced memory characteristics, both N_t_ and D_it_ were evaluated; the results are plotted in Figure 9.

The effective trapped charge density (N_t_) was estimated using Equation (1), evaluated at the sweep voltage at which the memory window saturated:

(1) $$N_{t}=\frac{C_{ox}\cdot \Delta V_{FB}}{q\cdot A}\;\left[{cm}^{-2}\right]$$

where C_ox_ is the accumulation-region capacitance, ΔV_FB_ is the memory window (the flat-band voltage difference between the program and erase states), q is the elementary charge, and A is the electrode area. It should be noted that N_t_ obtained in this way represents an effective trapped charge density under the specific voltage-sweep protocol used, rather than the total physical density of trap states in the material. Figure 9a presents N_t_ as a function of both DP-HfO_2_ thickness (bottom axis) and RP-HfO_2_ thickness (top axis), allowing direct comparison across all device configurations. The DPALD device exhibited the lowest N_t_ of 1.35 × 10^13^ cm^−2^, whereas the RPALD device showed a higher value of 2.11 × 10^13^ cm^−2^. N_t_ increased systematically with the RP interfacial fraction: the RP/DP/RP (1/8/1 nm) device showed 1.64 × 10^13^ cm^−2^, close to the DPALD value, whereas the (2/6/2), (3/4/3), and (4/2/4 nm) devices reached 2.03, 2.26, and 2.21 × 10^13^ cm^−2^, respectively, comparable to or slightly above the RPALD reference. The lower N_t_ observed in the DPALD device may be associated with interface damage related to the ion-assisted DP process, which could hinder efficient charge trapping and detrapping through the tunnel oxide, thereby potentially reducing the net effective trapped charge density despite the presence of crystalline-phase trap sites. As indicated by the dual *x*-axis in Figure 9a, N_t_ tended to decrease with increasing DPALD layer thickness, which is consistent with this interpretation. The slightly higher N_t_ of the modulated devices with thicker RP interfaces relative to the RPALD device may reflect the combined contribution of trap sites associated with the DPALD bulk layer and reduced interface defects associated with the RPALD interfacial layers. These N_t_ trends are consistent with the MW results in Figure 8, suggesting that inserting RPALD interfacial layers in the modulated RP/DP/RP stack recovers the effective trapped charge density toward the RPALD level.

The D_it_ distributions were extracted from the high-frequency (1 MHz) C–V curves using a voltage-difference method based on the stretch-out of the measured C–V curve relative to an ideal (trap-free) curve [38], which relates the stretch-out of the C–V characteristics to the interface trap density as a function of surface potential. The surface potential ψ was obtained by integrating the measured C–V curve (Equation (3)), with the integration constant D set to zero at flat band, and the interface trap density was then evaluated from Equation (2):

(2) $$D_{it}=\frac{C_{ox}}{q\cdot A}\left(\frac{\Delta V}{\Delta E}\right)\left[{eV}^{-1}{cm}^{-2}\right](\Delta V=\Delta V_{D_{it}}-{\Delta V}_{\text{ideal}})$$

(3) $$\psi =\int_{V_{G1}}^{V_{G2}}\left(1-\frac{C}{C_{ox}}\right)\;dV_{G}+D\;(D=0)$$

where C_ox_ is the oxide capacitance, q is the elementary charge, ΔV = ΔV_Dit_ − ΔV_ideal_ is the stretch-out of the measured C–V curve relative to the ideal (trap-free) curve, i.e., the difference between their gate-voltage spans over the same capacitance range, and ΔE is the corresponding surface-potential variation. Figure 9b shows the D_it_ distributions for four representative conditions (DPALD, RPALD, and the modulated 2/6/2 nm and 3/4/3 nm structures). All devices exhibited a U-shaped D_it_ distribution, with higher values near the band edge (E − E_i_ ≈ −0.45 eV) and lower values near midgap, consistent with the typical response of the Si/Al_2_O_3_ semiconductor interface. The DPALD device exhibited the highest D_it_ across the entire energy range, which may indicate that ion bombardment during the DP process introduces additional interface defects that reduce charge trapping efficiency. Both the RPALD and RP/DP/RP (3/4/3 nm) devices maintained comparably low D_it_ throughout the energy range. At the minimum point near midgap, the RP/DP/RP (3/4/3 nm) device showed a D_it_ of 2.5 × 10^12^ eV^−1^cm^−2^, among the lowest of the evaluated devices. It should be noted that the high-frequency C–V response is primarily sensitive to the Si/Al_2_O_3_ interface and to near-interface/border traps, and does not independently resolve the individual HfO_2_/Al_2_O_3_ interfaces. Taken together, these results suggest that incorporating RPALD interfacial layers in the modulated RP/DP/RP stack improves the interfacial quality relative to the DPALD device while recovering an effective trapped charge density comparable to that of the RPALD reference.

**Figure 10:**
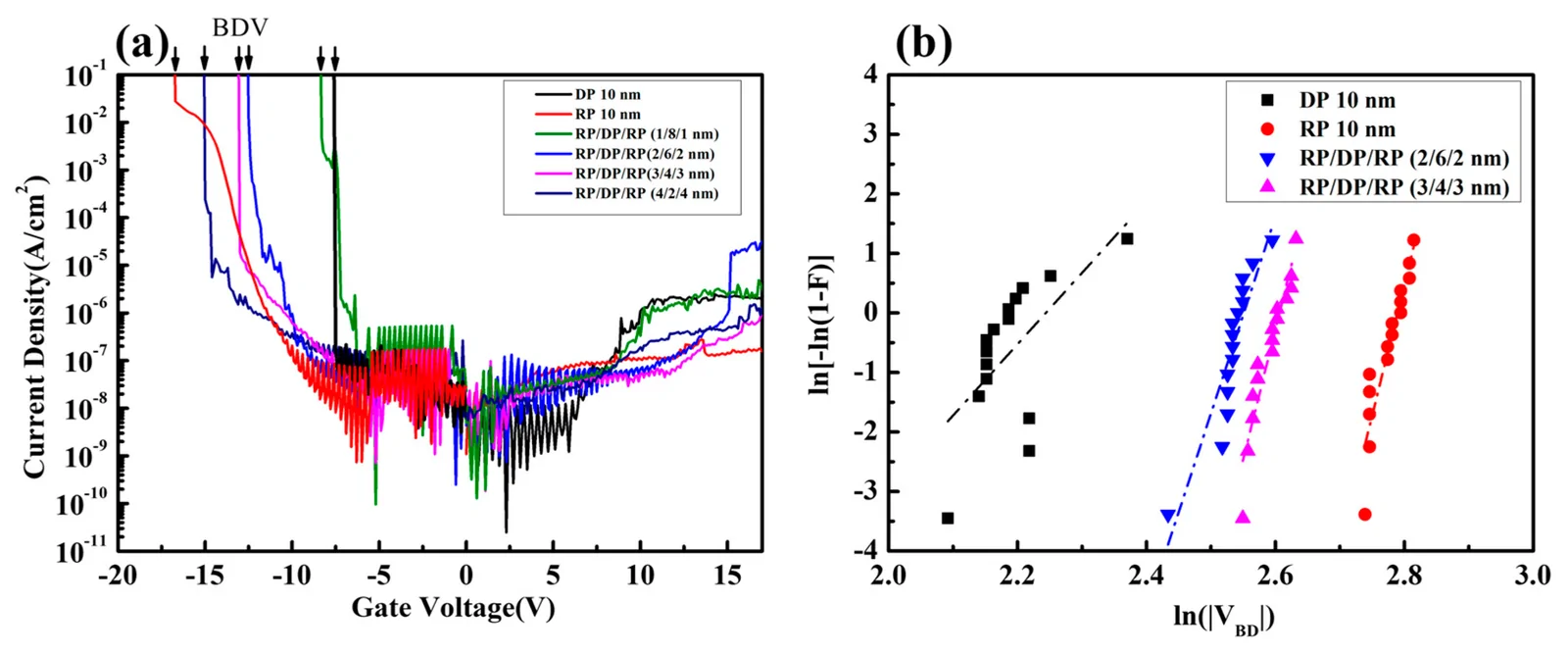
(**a**) J–V characteristics and (**b**) Weibull distribution of dielectric BDVs for HfO_2_ CTM devices fabricated using DPALD, RPALD, and modulated RP/DP/RP structures.

The current-density–gate-voltage (J–V) characteristics of HfO_2_ CTM devices fabricated using DPALD, RPALD, and modulated stacking sequences are shown in Figure 10a. The J–V characteristics were measured under both forward and reverse gate-bias conditions.

The DPALD device exhibited a breakdown voltage (BDV) of approximately −8.4 V. In contrast, the RPALD device showed the highest |BDV| of approximately −16.8 V and maintained a stable leakage current density of ≈3 × 10^−6^ A/cm^2^ throughout the positive-bias sweep, which is consistent with improved dielectric integrity. The modulated RP/DP/RP devices showed a systematic dependence of BDV on the RP interfacial thickness, with values of approximately −7.6, −12.6, −13.1, and −15.1 V for the (1/8/1), (2/6/2), (3/4/3), and (4/2/4 nm) structures, respectively; the |BDV| increased with the RP interfacial fraction and progressively approached that of the RPALD reference. The modulated devices also maintained low leakage current densities in the forward-bias region, suggesting that the incorporation of RPALD interfacial layers may help reduce charge leakage.

To statistically evaluate the reliability of the dielectric breakdown characteristics, Weibull distribution analysis [39] was performed based on 15 devices per condition; the results for four representative conditions (DPALD, RPALD, and the modulated 2/6/2 nm and 3/4/3 nm structures) are presented in Figure 10b. The DPALD device population is located in the lowest ln(|V_BD_|) ≈ 2.09–2.37 region (corresponding to |V_BD_| ≈ 8.1–10.7 V), the modulated RP/DP/RP (2/6/2 nm) and (3/4/3 nm) populations occupy successively higher ranges of ln(|V_BD_|) ≈ 2.43–2.60 and ≈2.55–2.63 (|V_BD_| ≈ 11.4–13.4 and ≈12.8–13.9 V), respectively, and the RPALD population is located at the highest ln(|V_BD_|) ≈ 2.74–2.81 range (|V_BD_| ≈ 15.5–16.7 V), indicating the improvement in BDV. The extracted Weibull slope (β) for the DPALD device was the lowest at 14.47, indicating relatively large device-to-device variability, while the RPALD device exhibited the highest β of 44.02, which corresponds to a narrower breakdown distribution. The modulated RP/DP/RP devices also showed substantially higher Weibull slopes, remaining comparably high across the series (e.g., β = 43.84 for the 3/4/3 nm device). These results suggest that the introduction of RPALD interfacial layers in the modulated RP/DP/RP scheme may reduce leakage associated with grain boundaries and improve dielectric breakdown characteristics, while also contributing to device-to-device uniformity.

**Figure 11:**
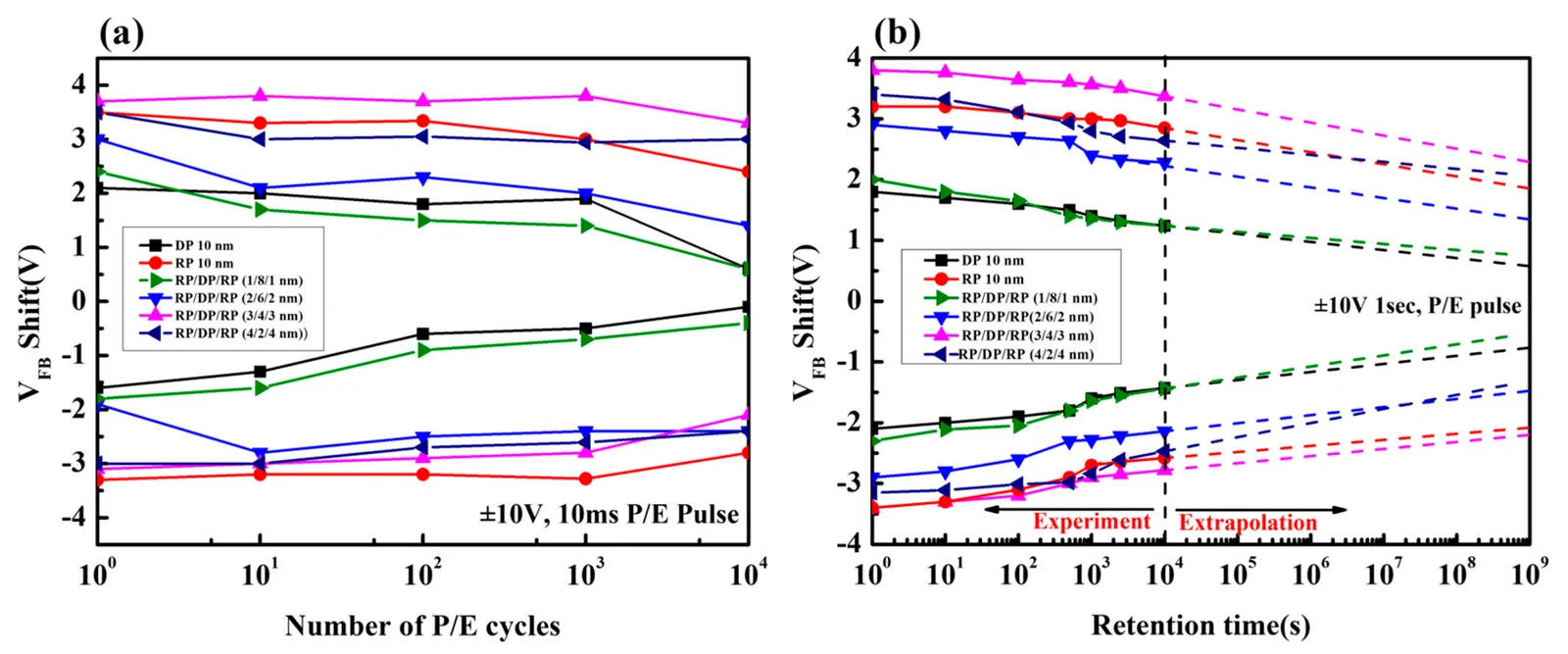
Reliability analysis of HfO_2_ CTM devices: (**a**) endurance characteristics and (**b**) memory retention.

The endurance characteristics of the HfO_2_ CTM devices were evaluated over 10^4^ program/erase (P/E) cycles, as shown in Figure 11a. P/E cycling was performed using a pulse width of 10 ms and a stress voltage of ±10 V. The initial MW of the RP/DP/RP (3/4/3 nm) device was the widest among all devices, with V_FB_ shifts of approximately +3.7 V and −3.1 V for the program and erase states, respectively, while the DPALD device exhibited a comparatively narrower initial window with V_FB_ shifts of approximately +2.1 V and −1.6 V. The RP/DP/RP (2/6/2 nm) device exhibited an intermediate initial window, with V_FB_ shifts of approximately +3.0 V and −1.9 V. This pulsed initial window is smaller than the quasi-static C–V memory window (e.g., 9.3 V for the 3/4/3 nm device). This is because the two are measured under different programming protocols, pulse widths, and charge-injection conditions. As the number of repetitive field stress cycles increased, distinct differences in endurance behavior were observed depending on the plasma modulation scheme employed.

The DPALD device exhibited a pronounced narrowing of the MW over cycling—its program-state V_FB_ shift decreasing from +2.1 V to +0.6 V and its erase-state shift from −1.6 V to −0.1 V after 10^4^ cycles—which may be associated with the progressive accumulation of interface defects under repetitive electrical stress and possible charge detrapping through grain-boundary pathways. In contrast, the RPALD device maintained relatively stable V_FB_ shifts, changing only from +3.5 V to +2.4 V (program) and from −3.3 V to −2.8 V (erase) after 10^4^ cycles. Similarly, the RP/DP/RP (3/4/3 nm) device retained a stable MW, with program- and erase-state V_FB_ shifts of approximately +3.3 V and −2.1 V after 10^4^ cycles, showing endurance behavior comparable to that of the RPALD device. The RP/DP/RP (1/8/1 nm) device showed a program-state V_FB_ shift decreasing from +2.4 V to +0.6 V and an erase-state shift from −1.8 V to −0.4 V after 10^4^ cycles, remaining closer to the DPALD behavior, whereas the RP/DP/RP (4/2/4 nm) device exhibited more stable shifts, from +3.5 V to +3.0 V (program) and from −3.0 V to −2.4 V (erase), closely approaching the RPALD behavior. These results suggest that the incorporation of RPALD interfacial layers may mitigate degradation under repetitive electrical stress, contributing to more stable endurance behavior within the tested range of 10^4^ cycles. A more comprehensive assessment over an extended number of cycles would be required to further establish the long-term switching reliability.

The long-term charge retention characteristics measured at 25 °C are presented in Figure 11b. P/E pulses were applied with a pulse width of 1 s and a stress voltage of ±10 V, and the V_FB_ values were measured up to 10^4^ s; the behavior beyond this range was estimated by extrapolation to identify the overall retention trend. The DPALD device exhibited a pronounced decay in the V_FB_ values of both the program and erase states within the measured range, and the extrapolated trend suggests that its MW may be substantially reduced over extended operation. In contrast, devices incorporating RPALD interfacial layers exhibited more stable charge retention characteristics. The RP/DP/RP (2/6/2 nm) device also showed relatively stable retention characteristics, although with a slightly smaller projected MW than that of the 3/4/3 nm device, and the same trend held across the modulated structures, with the (4/2/4 nm) device close to the RPALD behavior, projected to reach program- and erase-state V_FB_ shifts of approximately +2.58 V and −1.54 V at the 10-year mark, and the (1/8/1 nm) device closer to that of DPALD, projected to reach approximately +0.97 V and −0.9 V at the 10-year mark. In particular, the extrapolated retention trend of the RP/DP/RP (3/4/3 nm) device projects program- and erase-state V_FB_ shifts of approximately +3.37 V and −2.6 V, respectively, at the 10-year mark, suggesting long-term charge retention comparable to that of the RPALD device. These results suggest that the incorporation of RPALD interfacial layers within the modulated RP/DP/RP stack may help preserve charge-storage stability. The projected values beyond 10^4^ s represent an extrapolated trend rather than measured data; dedicated long-term retention testing and elevated-temperature evaluation would therefore be required to assess the actual long-term reliability of the devices [40].

Table 2 summarizes the electrical characteristics of the RP/DP/RP (3/4/3 nm) device alongside previously reported HfO_2_-based CTM structures; the device achieved a comparable MW while operating at a lower annealing temperature (500 °C) than most of the compared studies.

## 4. Conclusions

In this study, a modulated hybrid PEALD process employing an interface–bulk–interface (RP/DP/RP) stacking design was proposed to improve the performance and reliability of HfO_2_-based CTM devices, and the electrical characteristics were systematically evaluated as a function of layer thickness combination.

Thin film characterization showed that DPALD provided a high GPC of 1.05 Å/cycle and appeared to contribute bulk trap sites through crystalline phase formation; however, it was also accompanied by increased surface roughness and grain-boundary-related leakage paths consistent with enhanced crystallization. RPALD exhibited a lower GPC of 0.74 Å/cycle and showed improved interfacial quality through reduced crystallization and reduced surface roughness. Across the RP/DP/RP structures, the device characteristics varied systematically with the RP interfacial fraction, shifting from DPALD-like to RPALD-like behavior. The modulated devices improved upon the DPALD reference and reached values comparable to the RPALD reference; for example, the RP/DP/RP (3/4/3 nm) structure yielded an MW of 9.3 V under ±10 V operation, higher than the DPALD device (5.0 V) and comparable to the RPALD device (8.7 V) within the device-to-device variation. Trap analysis showed that the modulated structures with sufficient RP interfacial thickness combined a high effective trapped charge density (N_t_) comparable to that of RPALD with a low near-interface trap density (D_it_) at the Si/Al_2_O_3_ interface, indicating a favorable combination of a high bulk trap contribution and a low interfacial defect density. Dielectric breakdown analysis showed that the RP/DP/RP (3/4/3 nm) device had a BDV of approximately −13.1 V and a Weibull slope (β) of 43.84, which corresponds to a relatively narrow breakdown distribution. Reliability evaluation further showed stable MW retention over 10^4^ P/E cycles within the tested range, and linear extrapolation of the charge retention data projected program- and erase-state V_FB_ shifts of approximately +3.37 V and −2.6 V, respectively, at the 10-year mark, although this projection is based on short-term measurements and requires longer-term experimental verification.

These results suggest that the RP/DP/RP process design provides a process-level route to independently control interfacial quality and bulk trap characteristics without modifying the material composition. By applying RPALD at the interfacial regions and DPALD in the bulk, this approach mitigates the trade-off between bulk trap density and interfacial quality inherent to each single-mode process, and may offer a useful pathway toward reliable HfO_2_-based CTM devices. It should be noted, however, that only symmetric stacking combinations were examined and that the long-term retention was inferred from extrapolation; asymmetric structural studies, interface-sensitive analyses, extended reliability testing, and in situ plasma diagnostics remain important directions for future work.

## Figures and Tables

**Table 1:** Cycle allocation and thickness of the HfO_2_ CTLs used for the CTM devices. The number of RPALD and DPALD cycles for each region was calculated using the single-mode GPC values (0.74 Å/cycle for RPALD and 1.05 Å/cycle for DPALD) to reach the target sublayer thicknesses. Measured thicknesses correspond to the total HfO_2_ stack, obtained by spectroscopic ellipsometry.

Structure	RP Cycles (Bottom)	DP Cycles (Bulk)	RP Cycles (Top)	Total Cycles	Nominal Thickness (nm)	Measured Thickness (nm)
DP 10 nm	-	95	-	95	10	9.96
RP/DP/RP (1/8/1 nm)	14	76	14	104	10	9.54
RP/DP/RP (2/6/2 nm)	27	57	27	111	10	10.1
RP/DP/RP (3/4/3 nm)	41	38	41	120	10	9.99
RP/DP/RP (4/2/4 nm)	54	19	54	127	10	9.94
RP 10 nm	135	-	-	135	10	9.98

**Table 2:** Comparison summarizing representative HfO_2_-based CTM devices.

Structure (BO/CTL/TO)	CTL Thickness (nm)	Operating Voltage (V)	MW (V)	Annealing Temperature (°C)	References
Al_2_O_3_/HfO_2_ (RP/DP/RP ALD)/Al_2_O_3_	3/4/3	±10	9.3	500	This work
HfO_2_ (e-beam Evaporation)/SiO_2_	55	±10	5.1	800	[12]
Al_2_O_3_/HfO_2_ (RP/DP ALD)/SiO_X_	7/3	±10	10.1	400	[26]
Al_2_O_3_/HfO_2_ (ALD)/SiO_2_	9	±16	11.5	800	[41]

## Data Availability

Data are contained within the article.

## Abbreviations

The following abbreviations are used in this manuscript:

CTM	Charge trap memory
CTL	Charge trap layer
ALD	Atomic layer deposition
RPALD	Remote plasma ALD
DPALD	Direct plasma ALD
